# Brucella-associated hemophagocytic syndrome: case report of a potentially life-threatening condition and literature review

**DOI:** 10.3389/fimmu.2025.1592089

**Published:** 2025-07-25

**Authors:** Limei Shi, Bin Wang, Danping Peng, KaiYu Zhang, Yang Wang

**Affiliations:** Center of Infectious Disease and Pathogen Biology, Department of Infectious Diseases, The First Hospital of Jilin University, Changchun, China

**Keywords:** brucellosis, hemophagocytic syndrome, pancytopenia, diagnosis, anti-brucellosis therapy

## Abstract

Brucellosis is a highly contagious zoonotic disease characterized by a non-specific clinical presentation and complex disease progression and outcome. Hemophagocytic lymphohistiocytosis (HLH) is an abnormal immune response syndrome marked by potentially fatal cytokine storms. Brucella-associated HLH is exceedingly rare and associated with a high mortality rate. We report a case involving a 23-year-old male residing in a brucellosis-endemic region, with a documented history of exposure to cattle and sheep. He was admitted to the hospital presenting with fever and arthralgia. Laboratory tests indicated splenomegaly, pancytopenia, elevated serum aminotransferases and ferritin levels, as well as decreased fibrinogen levels. Blood and bone marrow cultures yielded negative results. The Brucella serum agglutination test returned a positive result (titer, 1:200). Bone marrow aspirate results revealed an increased number of hemophagocytes, and PET-CT scans demonstrated splenomegaly, suggesting hemophagocytic changes. Following a comprehensive exclusion of hematological malignancies and neoplastic diseases, the patient was diagnosed with probable Brucella infection complicated by secondary HLH. Standard anti-brucellosis therapy was initiated immediately upon hospital admission. Remarkably, significant clinical improvement was observed within 7 days of targeted antibiotic treatment, without the need for corticosteroid therapy. This case, when analyzed alongside a systematic review of 12 published HLH cases associated with brucellosis, underscores the importance of maintaining a heightened clinical suspicion for this life-threatening complication in endemic regions, which may facilitate earlier diagnosis and optimized antimicrobial management strategies.

## Introduction

*Brucella* spp. are facultative intracellular gram-negative pathogens responsible for brucellosis, a globally prevalent zoonotic disease transmitted through direct exposure to infected livestock or the consumption of unpasteurized dairy products. This disease imposes a substantial economic burden and presents ongoing public health challenges. While classical manifestations include undulant fever, sweats, and migratory polyarthralgia, severe complications may arise, such as hematological emergencies, including secondary HLH. HLH is a life-threatening hyperinflammatory syndrome characterized by dysregulated cytotoxic T-cell (CTL) and natural killer (NK) cell activity, as well as macrophage activation, rather than being a distinct disease entity (1, 2). Pathophysiologically, HLH manifests as a cytokine storm, which can culminate in multiorgan failure. Its clinical management remains challenging due to overlapping features with septicemia (3). Here, we present a diagnostically challenging case involving a 23-year-old male patient who exhibited fever, arthralgia, splenomegaly, and pancytopenia. The patient showed improvement following anti-brucellosis treatment, with no corticosteroid therapy administered. He was diagnosed with probable Brucella infection complicated by secondary HLH. The cases of Brucella infection complicated by HLH are exceedingly rare. This case underscores the importance of obtaining a comprehensive epidemiological history and conducting serological testing for brucellosis during the diagnostic process. It also highlights the necessity of initiating empirical anti-brucellosis therapy in patients with rapid disease progression when clinical suspicion is high. For brucellosis-associated HLH, bone marrow biopsy and PET-CT imaging are effective tools that expedite diagnostic evaluation and help differentiate it from other underlying conditions.

## Case report

### Clinical manifestation

A 23-year-old male from Inner Mongolia, China, presented to our hospital with persistent joint pain lasting one year and an acute onset of fever accompanied by a cough over the past three days. The patient initially developed migratory polyarthralgia affecting large joints (knees and ankles) for one year prior to admission, which had not been medically evaluated. Three days before admission, he experienced a sudden onset of fever (peak temperature of 38.8°C) accompanied by chills and a paroxysmal non-productive cough. Initial treatment at a local clinic included intravenous cephalosporin therapy (2.0 g, QD, i.v.). Within a few hours of the initial treatment, he developed facial edema, conjunctival hemorrhage, and epigastric pain. Despite continued treatment with cephalosporin, his symptoms did not improve, prompting his referral to our institution. During the outpatient visit to our hospital, the patient’s laboratory tests indicated pancytopenia, increased levels of aminotransferases, elevated ultrasensitive C-reactive protein, and decreased fibrinogen. Upon admission, the physical examination revealed facial edema, bilateral conjunctival congestion, enlarged cervical lymph nodes, abdominal tenderness without rebound pain, and muscle tension. The patient resided in Inner Mongolia and kept cows and sheep at home, with a history of feeding them. Notably, there was an incident of abortion in the cows and sheep one month prior to admission. The patient did not wear gloves while handling the fetus. There was no history of tuberculosis, recent travel, blood transfusions, alcohol consumption, smoking, or other relevant factors. Additionally, there was no family history of similar conditions.

### Diagnostics and examinations

**Figure 1:**
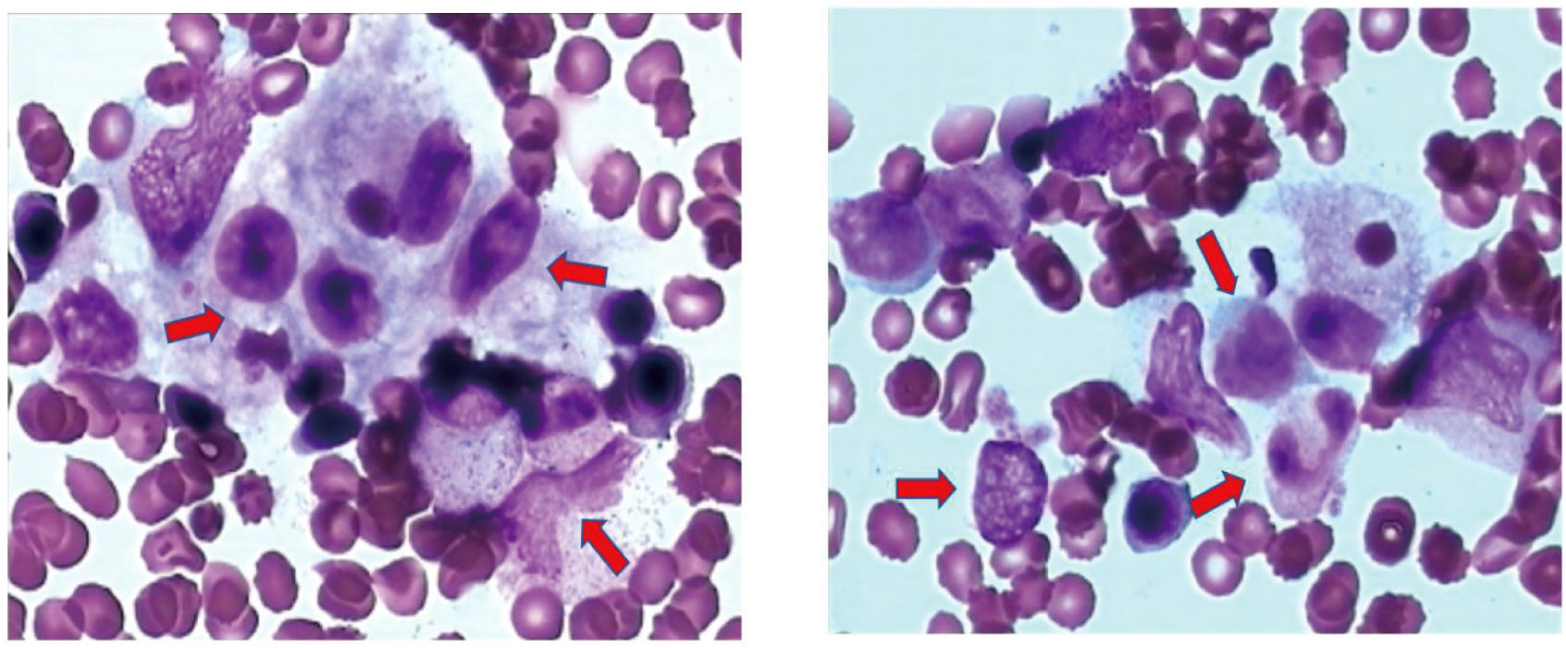
Bone marrow smear. Histiocytes and phagocytes are easily visible. Histiocytes accounted for 2.5% and phagocytic cells accounted for 2.5%, and phagocytic erythrocytes were visible. Arrows indicate hemophagocytes. Hemophagocytosis refers to the pathological process where macrophages (a type of immune cell) engulf and destroy other blood cells, such as red blood cells, white blood cells, or platelets.

**Figure 2:**
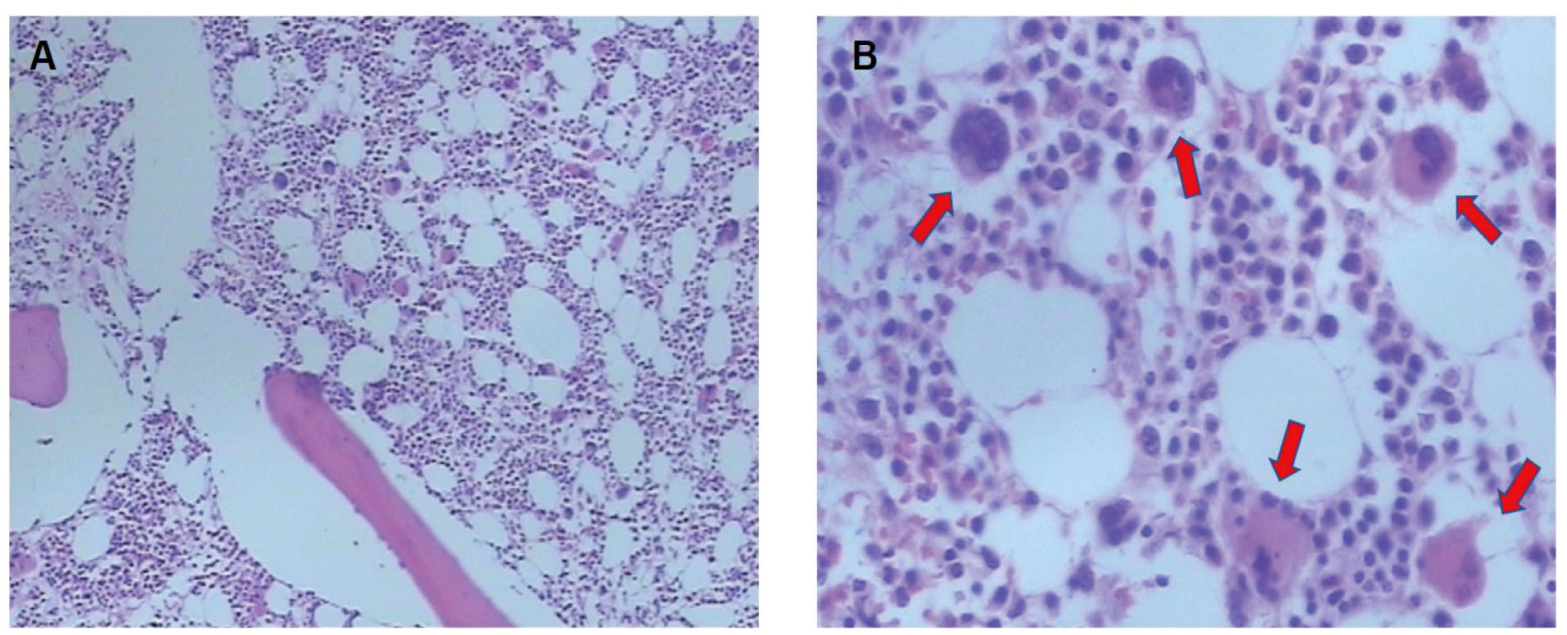
Bone marrow aspiration biopsy. **(A)** Bone marrow proliferation is markedly active, with an increased percentage of red lineage. **(B)** The arrows point to the easily visible megakaryocytes.

**Figure 3:**
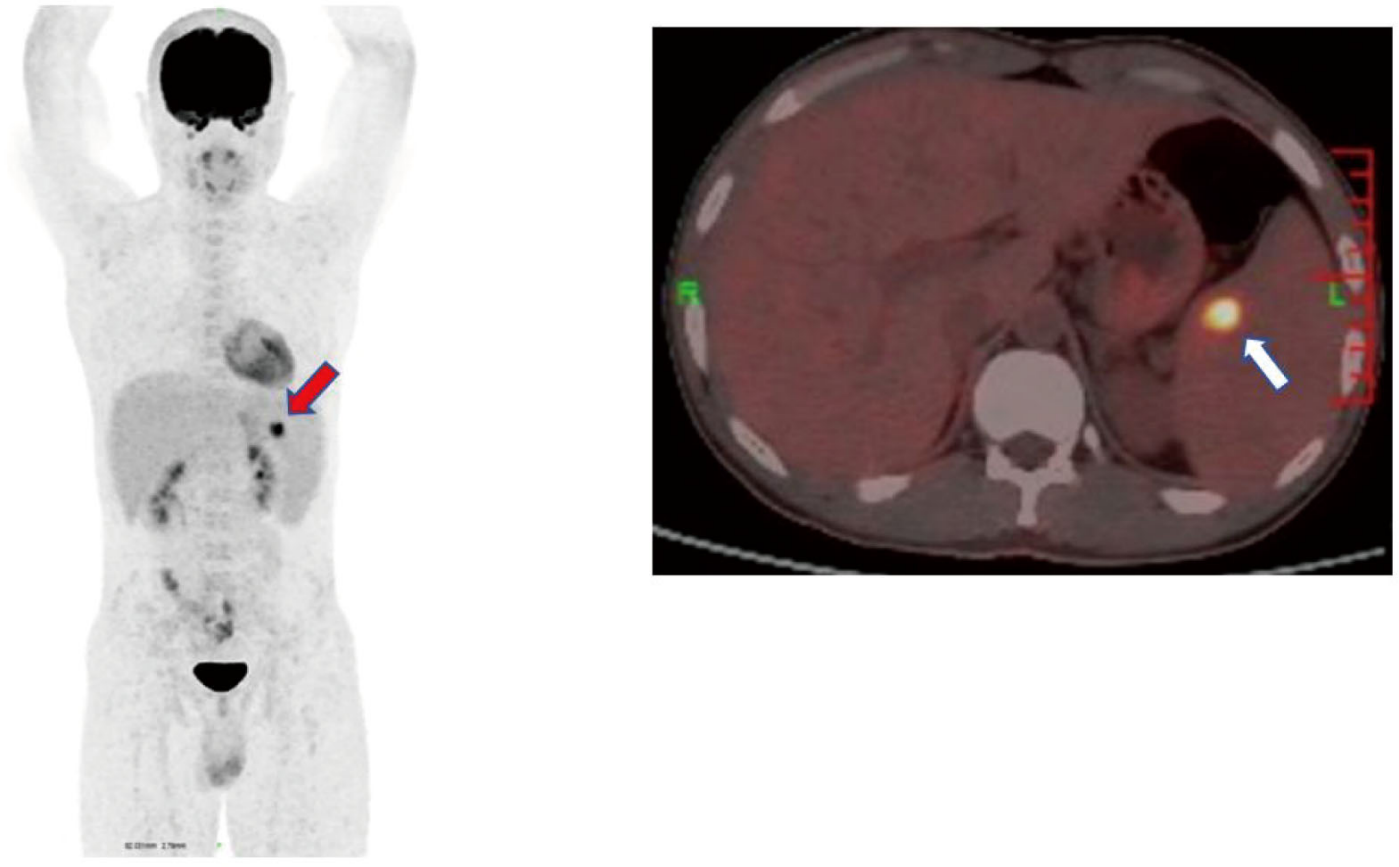
PET-CT whole-body imaging splenomegaly. There are focal hypermetabolic nodules inside, which cannot be ruled out as hemophagocytic changes. Both red and white arrows indicate spleens with hypermetabolic nodules. .

The patient was admitted to the hospital with a fever of unknown origin. Major laboratory tests conducted post-admission revealed pancytopenia, elevated aminotransferases, elevated C-reactive protein (CRP), and decreased fibrinogen (**Table 1**). To determine the cause of the fever, we performed a series of etiological tests, including procalcitonin, antibodies against the epidemic hemorrhagic fever virus, cytomegalovirus nucleic acid quantification and antibodies, Epstein-Barr virus (EBV) nucleic acid quantification and antibodies, as well as a tuberculosis interferon-gamma release assay and a tuberculin skin test, all of which yielded negative results. We also tested for rheumatic immunology markers, including rheumatological factor, anti-cyclic citrullinated peptide antibodies, and antinuclear antibodies, all of which were negative. Blood cultures also returned negative. Given the patient’s epidemiological history and clinical manifestations, including fever, arthralgia, splenomegaly, and lymphadenopathy, there was a strong suspicion of Brucella infection. Further testing using the Brucella serum agglutination test (SAT) confirmed this suspicion, yielding a positive result at a titer of 1:200. Due to the patient’s severe condition, characterized by pancytopenia and elevated ferritin levels upon admission, we conducted bone marrow aspiration, tumor marker testing, and PET-CT to rule out other hematological or neoplastic diseases. Simultaneously, a bone marrow culture was performed. The bone marrow smear exhibited myeloproliferative activity, with histiocytes and phagocytes clearly visible; histiocytes accounted for 2.5%, and phagocytes also accounted for 2.5% (**Figure 1**). The bone marrow biopsy revealed markedly active myeloproliferative activity with an elevated red lineage ratio, and megakaryocytes were readily observed (**Figure 2**). However, the bone marrow culture returned negative results. Additionally, the PET-CT scan indicated splenomegaly, characterized by hypermetabolic nodules, and did not exclude hemophagocytic changes (**Figure 3**). Furthermore, abdominal CT demonstrated splenomegaly, edema of the gallbladder wall, and ascites. Lung CT revealed bronchitis in the right middle lobe, inflammation in the left lingula lobe, scattered inflammation in both lower lobes, and bilateral pleural effusion. Abdominal ultrasound confirmed splenomegaly and identified an intrasplenic, flaky, hypoechoic lesion. Cardiac ultrasound indicated a small pericardial effusion. Following the hematologist’s advice, we monitored for HLH. Soluble CD25 was 20984 pg/ml (normal range: <6,400), and NK cell activity was recorded at 18.57% (normal range: 47.6%-76.8%). Consequently, a probable Brucella infection with secondary HLH was suspected.

**Table 1:** Summary of the laboratory tests in the patient.

Inspection	D1	D2	D3	D4	D8	Normal range
WBC	1.71	1.66	1.9	2.66	4.17	3.5-9.5 10^^9^/L
HGB	109	99	96	92	103	130–175 g/L
RBC	3.68	3.39	3.28	3.16	3.54	4.3-5.8 10^^12^/L
PLT	27	44	57	88	299	125-350 10^^9^/L
AST	286.2				63.6	15–40 U/L
ALT	122.2				31.2	9–50 U/L
Albumin	28.9				30.6	40–55 g/L
CRP	87.44				<1	0–1 mg/L
D-dimer	14.38	7.85				0–05 mg/L
Fibrinogen	1.62	1.46		1.44	1.06	1.8-4.0 g/L
LDH	630				390	120–250 U/L
Cholesterol			1.52		3.84	2.6–6 mmol/L
Triglycerides			2.29		2.15	0.28-1.8 mmol/L
Ferritin			562.4		308.3	23.9-336.2 µg/L

WBC, white blood cell; HGB, Hemoglobin; PLT, Platelet; AST, Aspartate transferase; ALT, Alanine transferases; CRP, C-reactive protein; LDH, Lactate dehydrogenase

### Treatment and regression

Initially, an anti-brucellosis treatment regimen consisting of doxycycline combined with amikacin was administered. However, due to elevated transaminase levels, rifampicin was temporarily withheld, and hepatoprotective therapy was initiated. On the fourth day of anti-brucellosis treatment, the patient’s fever showed improvement. By the eighth day, the patient was afebrile, blood counts had improved, aminotransferase levels had decreased, ferritin levels returned to normal, and triglyceride levels had diminished (**Table 1**). Rifampicin was subsequently added to the treatment plan. The patient was discharged on the ninth day of treatment. Following discharge, the patient continued an oral regimen of doxycycline (0.1 g, BID, p.o.) combined with rifampicin (0.6 g, QD, p.o.) for six weeks, along with intravenous amikacin (0.6 g, QD, i.v.) for two weeks. After six weeks of consistent treatment, the patient was followed up in the outpatient clinic and achieved complete recovery.

## Discussion

In this case, the patient was admitted to the hospital with a persistent fever and migratory polyarthralgia as the initial symptoms. The patient exhibited typical epidemiological characteristics of Brucella infection, including: long-term residence in Inner Mongolia, a brucellosis-endemic region, and a clear history of occupational exposure to cattle and sheep. Therefore, brucellosis was prioritized as the initial differential diagnosis. However, the patient also presented with pancytopenia and elevated inflammatory markers, necessitating prompt intervention to prevent clinical deterioration. We identified the following potential conditions in the early stages: (1) hematologic malignancies, such as acute leukemia or myelodysplastic syndrome; (2) lymphoproliferative disorders, such as lymphoma-related HLH; and (3) autoimmune diseases, such as systemic lupus erythematosus. Following the WHO guidelines for the management of brucellosis, we initiated empiric dual antimicrobial therapy immediately upon hospitalization, alongside confirmatory tests, including serum tube agglutination tests and blood cultures. In this high-risk epidemiological context, this aggressive treatment approach effectively balances diagnostic investigations with timely antimicrobial intervention.

Brucellosis is a prevalent and often overlooked zoonotic disease. In recent years, it has reemerged in various regions of China, particularly in Inner Mongolia, where animal husbandry is the predominant industry (4). The disease can affect individuals of any age group. Recently, the clinical symptoms have become increasingly atypical, with some patients lacking a clear epidemiological history or presenting with negative blood cultures. Consequently, diagnosis has become progressively challenging (5). Brucellosis can lead to a range of complications. When the hematologic system is involved, it may present with various manifestations, including anemia, leukopenia, thrombocytopenia, and pancytopenia. Our patient exhibited pancytopenia upon admission. This condition may be associated with factors such as infection, hypersplenism, and bone marrow suppression. In rare cases, severe pancytopenia can be attributed to diffuse intravascular coagulation, increased hemophagocytes, or immune-mediated cell destruction (6, 7). The association between brucellosis and pancytopenia may be attributed to HLH. HLH consists of both primary and secondary components. Primary HLH (pHLH) is a hereditary condition that is more commonly observed in children, whereas secondary HLH (sHLH) predominantly affects adults. sHLH is characterized by immune overactivation and dysregulation triggered by various factors, including infections, tumors, and autoimmune diseases. The Epstein-Barr virus is the most frequently identified pathogen associated with infections, while Brucella is relatively rare (8). An innate immune response triggered by toll-like receptors (TLRs) in response to an infectious agent is one of the mechanisms leading to HLH. *Brucella* infection led to a significant change in the composition of peripheral immune cells, and inflammation was a key feature of brucellosis. The highest inflammatory state can be observed in acute patients. Excessive production of cytokines, such as interleukin-6 (IL-6), IL-1β, and IFN-γ, can lead to life-threatening complications (9, 10). Additionally, some patients may possess a genetic variant that hinders the resolution of the immune response, leading to excessive inflammation (11). There is a notable overlap of symptoms between brucellosis and sHLH, including persistent fever, hepatosplenomegaly, and pancytopenia. These symptoms are also prevalent in other infectious diseases, hematological disorders, and tumors, which can complicate the diagnostic process and increase the risk of misdiagnosis (12).

In the process of diagnosing brucellosis, our patient presents a clear epidemiological history, with clinical manifestations consistent with those of brucellosis, thereby raising a strong suspicion of Brucella infection. Additionally, SAT titers showed a notable increase following admission, reaching 1:200. Titers of 1:100 or higher, with at least 50% agglutination, were classified as positive. SAT titers of 1:160 or more, when aligned with the clinical symptoms, are considered diagnostic. While SAT tests are conducted as a standalone assessment, they are frequently utilized as the main diagnostic standard in clinical settings when paired with exposure history and symptoms. Nevertheless, using several serological tests can enhance the accuracy of the diagnosis (13–15). To enhance the specificity of the SAT, a cutoff of 1:320 has been recommended for the serodiagnosis of brucellosis in endemic regions. However, raising the threshold may reduce sensitivity and compromise the accuracy of the diagnosis. Simultaneously, both blood and bone marrow cultures returned negative results. The reasons for these negative results may include a short incubation period for Brucella, the presence of a chronic infection, or prior antibiotic use. This suggests a high likelihood of brucellosis (16, 17) (18, 19). While positive blood cultures remain the gold standard for confirming brucellosis, their sensitivity ranges from 50% to 90%, depending on the disease stage, Brucella species, and technical factors (13, 20). Bone marrow cultures demonstrate a marginally higher but still suboptimal yield, and their sensitivity remains a subject of debate. Eduardo Gotuzzo et al. found that among 50 patients diagnosed with Brucella infection through culture testing, 46 patients (92%) had positive bone marrow cultures, while 35 patients (70%) had positive blood cultures (21). Mantur et al. reported that Brucella was isolated from bone marrow cultures in 85 (82.5%) of 103 patients, whereas blood cultures detected it in only 47 (45.6%) (22). However, Asem Shehabi et al. found that blood culture (44.4%) was more sensitive than bone marrow culture (27.7%) (23). Following the HLH-2004 diagnostic criteria: (1) fever; (2) splenomegaly; (3) cytopenias; (4) hypertriglyceridemia and/or hypofibrinogenemia; (5) hemophagocytosis in bone marrow or spleen or lymph nodes, no evidence of malignancy; (6) low or no NK cell activity; (7) ferritin ≥500 μg/L; (8) sCD25 (ie, soluble IL-2 receptor) ≥2400 U/ml. Our patients fulfilled all 8 criteria. Thus, the diagnosis of HLH can be definitively made. The comprehensive exclusion of alternative etiologies, including viral, autoimmune, hematologic, and neoplastic disorders, further strengthens the diagnostic validity (11).

A systematic literature review identified 12 previously reported cases of brucellosis complicated by secondary HLH (**Table 2**). The cohort consisted of 12 patients aged 25 to 75 years, with a balanced male-to-female ratio of 1:1. Epidemiological exposure was documented in 11/12 cases, including occupational or direct animal contact (6 cases: livestock farming or sheepherding), dietary exposure (2 cases: consumption of unpasteurized dairy), and recent travel to brucellosis-endemic regions (1 case). All these patients were admitted to the hospital with a fever of unknown origin, similar to our patient. Blood cultures were performed in all 12 cases of brucellosis, and three of these patients underwent additional bone marrow cultures. Brucella species were isolated in 9 blood cultures. Among the 3 patients with negative blood cultures, bone marrow culture was performed in one case, which also showed negative results, but the Brucella serology was positive. All 12 cases met the HLH-2004 diagnostic criteria. Each patient presented with fever, pancytopenia, and hyperferritinemia. Bone marrow aspiration was conducted in all patients. However, only three patients were tested for NK cell activity and soluble CD25 (sCD25). Notably, one case showed no evidence of hemophagocytosis on the bone marrow smear and lacked both NK cell activity and sCD25 testing. Given that fever and splenomegaly are common manifestations of brucellosis and also key diagnostic criteria for HLH, a comprehensive diagnostic evaluation is strongly recommended for patients with brucellosis complicated by HLH. In the cases reported here, the diagnostic tests for HLH included assessment of NK cell activity, sCD25 levels, and even PET-CT imaging, providing robust justification for the diagnosis.

**Table 2:** Case report and summary of brucellosis complicated with hemophagocytic syndrome.

Case	Gender	Age	Epidemiological history	Clinical characteristic	Inspection			Treatment	Outcome	Reference
					General examination	Culture	NK cell activity and sCD25 testing			
1	Female	25	history of drinking unpasteurized dairy milk	high-grade continuous fever,	cytopenia, increased hemophagocytes in bone marrow, elevated ferritin and aminotransferases, hepatomegaly, COVID PCR is positive, Dengue NS1 positive	blood cultures tested positive for Brucella, bone marrow culture unknown	NA	hormonal, meropenem, and anti-brucella therapy	on the 7th day of treatment, fever subsided, and stabilized	(24)
2	Male	32	history of consumption of fresh milk products	fever, sweating, and fatigue	splenomegaly, cytopenia, elevated serum transaminases, triglycerides, and ferritin, aminotransferases and increased hemophagocytes in bone marrow, Brucella IgG and IgM positive, Coombs anti-Brucella test titer was 1/320.	blood cultures were negative	NA	anti-brucella therapy	no fever on day 3 of treatment, complete remission after 6 weeks	(25)
3	Male	37	history of contact with sheep	dizziness, fatigue, nausea, vomiting, and joint pain, accompanied by a fever	cytopenia, hypofibrinogenemia, elevated ferritin, increased hemophagocytes in bone marrow, positive Brucella immunoglobulin G (IgG) antibodies, positive Brucella agglutination test 1:200 ++, and positive Brucella tiger red plate agglutination test (+)	blood cultures were negative	elevated sCD25, decreased NK cell activity	dexamethasone (17 mg/day) and etoposide (250 mg/day) combined with anti-brucella therapy	he was stable on the 12th day of treatment and recovered after 8 weeks	(26)
4	Female	45	history of animal slaughter	fever, skin rash	cytopenia, hepatosplenomegaly, increased hemophagocytes in bone marrow, elevated ferritin, Wright’s agglutination and 2-ME tests were positive	blood and bone marrow cultures are negative	NA	hydrocortisone and anti-brucella therapy	blood counts rose after 3 days of treatment, and the condition gradually recovered	(27)
5	Female	39	no clear epidemiological history	fatigue, anorexia, lassitude and mood disturbance, fever	splenomegaly, elevated ferritin and aminotransferases, cytopenia, increased hemophagocytes in the bone marrow, ovarian vein thrombosis	blood cultures tested positive for Brucella	NA	anti-brucella therapy	the patient’s condition and mood improved on treatment. ferritin levels and blood counts returned to normal	(28)
6	Female	41	history of a recent trip to the area with well- developed animal husbandry	fever, decreased appetite	increased hemophagocytes in bone marrow, cytopenia	blood cultures tested positive for Brucella	NA	anti-brucella therapy	the patient remained well at follow- up at 1 year	(29)
7	Female	50	history of sheep exposure	prolonged fever	hepatosplenomegaly, elevated ferritin, aminotransferases and triglycerides, cytopenia, and bone marrow biopsy showed a few histiocytes without any hemophagocytes or malignant cells seen.	blood cultures tested positive for Brucella	NA	anti-brucella therapy	blood counts normalized after 3 weeks of treatment. complete recovery after 6 weeks	(30)
8	Male	53	history of sheep exposure	abdominal pain, and prolonged fever	cytopenia, elevated ferritin and triglycerides, and increased hemophagocytes in bone marrow, elevated tumor markers (CEA, CYFRA21-1, NSE, CA19-9), and whole-body PET-CT scans ruled out tumor	blood cultures tested positive for Brucella	elevated sCD25, decreased NK cell activity	anti-brucella therapy	after 5 days of treatment, the temperature dropped and the blood count rose	(31)
9	Male	60	history of sheep exposure	fevers, chills, night sweats, and a 50-to-20-pound weight loss, decreased appetite	cytopenia, elevated ferritin, aminotransferases and triglycerides, fibrinogen is low, and bone marrow biopsy showing hemophagocytic, Brucella antibody agglutination test positive, Brucella IgG and IgM positive	blood cultures tested positive for Brucella	elevated sCD25, decreased NK cell activity	anti-brucella therapy and dexamethasone therapy	the patient’s fluctuating mentation and undulating fevers resolved completely	(32)
10	Male	53	history of sheep exposure	fatigue, arthralgia and fever	splenomegaly, cytopenia, hypertriglyceridemia, hypofibrinogenemia, and increased ferritin, increased hemophagocytes in bone marrow, Rose Bengal plate test was positive, standard tube agglutination (STA) test for brucellosis was 1:400	blood cultures tested positive for Brucella	NA	anti-brucella therapy and dexamethasone therapy	three weeks later, splenomegaly disappeared, and body temperature was normal. symptoms and abnormal laboratory findings were relieved after 4 weeks	(33)
11	Female	63	history of sheep exposure	unremitting fever and altered consciousness	splenomegaly, cytopenia, triglycerides and elevated ferritin, increased bone marrow phagocytosis, and blood next-generation sequencing was positive for Brucella	blood cultures tested positive for Brucella	NA	anti-brucella therapy	fever improved and general condition improved	(34)
12	Male	73	have a history of work as farmer	fever continuing, decreased appetite	hepatosplenomegaly, cytopenia, triglycerides, elevated aminotransferases and ferritin, positive Rose Bengal test for brucellosis	blood cultures tested positive for Brucella	NA	anti-brucella therapy	improvement in clinical condition and laboratory findings after 10 days of treatment	(35)

NA, not available.

Brucella-associated HLH progresses rapidly and has a high mortality rate. The prognosis for patients varies based on the severity of the underlying disease and the symptoms presented. Previous studies have indicated that HLH induced by intracellular infections typically does not necessitate HLH-94 therapy and is more effectively treated with specific antimicrobial therapy (36, 37). Among the 12 cases reviewed, 5 patients received a combination of corticosteroids and anti-brucellosis therapy, one patient underwent a regimen of corticosteroids, chemotherapy, and anti-brucellosis treatment, while six patients were managed exclusively with anti-brucellosis therapies (**Table 2**). Corticosteroids may be avoided in patients who respond quickly to antibiotic therapy and exhibit mild clinical manifestations of HLH. However, in severe cases, especially those complicated by other infections or organ failure. A short course of corticosteroids and/or intravenous immunoglobulin therapy should be considered to manage HLH. Although clear diagnostic criteria for brucellosis combined with HLH are lacking, patients exhibiting clinical features of Brucella-associated HLH require immediate empirical anti-brucellosis therapy. These findings suggest that controlling the primary infection may directly interrupt the pathological process of HLH. Timely intervention significantly reduces the risk of mortality, as delayed treatment can exacerbate organ damage mediated by inflammation storms.

## Limitations

As the main type of participants included were case reports, the quality of the studies was not high. This significantly reduces the robustness of the synthesis. Due to the limited data in this review, further similar cases are needed to elucidate this disease.

## Summary

Brucella infections cannot be overlooked. HLH associated with brucellosis typically affects children but is increasingly being diagnosed in adults. Brucellosis should be suspected in patients presenting with a fever of unknown origin and pancytopenia, regardless of their epidemiological history. Furthermore, HLH should be included in the differential diagnosis of severe complications arising from brucellosis. To ensure that patients receive effective treatment, clinicians urgently need to establish a clear definition of Brucella-associated HLH for accurate diagnosis. A prospective multicenter cohort study can be conducted to determine the incidence, risk factors, and early prediction models of Brucella-related HLH. Multi-omics analysis can reveal the key immune pathways involved in Brucella-induced HLH, and further investigation to inform targeted therapies.

## Data Availability

The original contributions presented in the study are included in the article/supplementary material. Further inquiries can be directed to the corresponding authors.
